# Connecting Smartphone and Wearable Fitness Tracker Data with a Nationally Used Electronic Health Record System for Diabetes Education to Facilitate Behavioral Goal Monitoring in Diabetes Care: Protocol for a Pragmatic Multi-Site Randomized Trial

**DOI:** 10.2196/10009

**Published:** 2018-04-02

**Authors:** Jing Wang, Deidra Carroll Coleman, Justin Kanter, Brad Ummer, Linda Siminerio

**Affiliations:** ^1^ Cizik School of Nursing The University of Texas Health Science Center at Houston Houston, TX United States; ^2^ University of Pittsburgh Medical Center Pittsburgh, PA United States; ^3^ Flipside Media, Inc Pittsburgh, PA United States; ^4^ School of Medicine University of Pittsburgh Pittsburgh, PA United States

**Keywords:** wearable devices, connected health, mobile health, diabetes, randomized clinical trial, goal setting, lifestyle intervention, electronic health record, self-monitoring, behavior modification

## Abstract

**Background:**

Mobile and wearable technology have been shown to be effective in improving diabetes self-management; however, integrating data from these technologies into clinical diabetes care to facilitate behavioral goal monitoring has not been explored.

**Objective:**

The objective of this paper is to report on a study protocol for a pragmatic multi-site trial along with the intervention components, including the detailed connected health interface. This interface was developed to integrate patient self-monitoring data collected from a wearable fitness tracker and its companion smartphone app to an electronic health record system for diabetes self-management education and support (DSMES) to facilitate behavioral goal monitoring.

**Methods:**

A 3-month multi-site pragmatic clinical trial was conducted with eligible patients with diabetes mellitus from DSMES programs. The Chronicle Diabetes system is currently freely available to diabetes educators through American Diabetes Association–recognized DSMES programs to set patient nutrition and physical activity goals. To integrate the goal-setting and self-monitoring intervention into the DSMES process, a connected interface in the Chronicle Diabetes system was developed. With the connected interface, patient self-monitoring information collected from smartphones and wearable fitness trackers can facilitate educators’ monitoring of patients’ adherence to their goals. Feasibility outcomes of the 3-month trial included hemoglobin A_1c_ levels, weight, and the usability of the connected system.

**Results:**

An interface designed to connect data from a wearable fitness tracker with a companion smartphone app for nutrition and physical activity self-monitoring into a diabetes education electronic health record system was successfully developed to enable diabetes educators to facilitate goal setting and monitoring. A total of 60 eligible patients with type 2 diabetes mellitus were randomized into either group 1) standard diabetes education or 2) standard education enhanced with the connected system. Data collection for the 3-month pragmatic trial is completed. Data analysis is in progress.

**Conclusions:**

If results of the pragmatic multi-site clinical trial show preliminary efficacy and usability of the connected system, a large-scale implementation trial will be conducted.

**Trial Registration:**

ClinicalTrials.gov NCT02664233; https://clinicaltrials.gov/ct2/show/NCT02664233 (Archived by WebCite at http://www.webcitation.org/6yDEwXHo5)

## Introduction

Obesity and type 2 diabetes mellitus (T2DM) are serious chronic illnesses in the US. Compared to a standard diabetes education, behavioral lifestyle interventions were found to be more effective in weight loss and diabetes control among overweight or obese patients with T2DM [1,2]. Self-monitoring and goal setting are two essential components of a behavioral lifestyle intervention [3,4]. Self-monitoring of healthy eating and physical activity play a key role in weight management and diabetes control in T2DM patients. Previous studies have shown that self-monitoring with an electronic diary is as effective as monitoring with a paper diary while being less burdensome and time-consuming [5,6]. Smartphones are now gaining attention for their use in facilitating patient self-monitoring of healthy eating and physical activity.

Research supports diabetes education as a cost-effective way to coordinate diabetes care. Diabetes self-management education and support (DSMES) programs located throughout the US are integrated within the existing health care system. Thus, DSMES programs present an ideal setting for testing the implementation of an evidence-based self-monitoring intervention. Chronicle Diabetes is a Web-based electronic health record (EHR) system designed to facilitate behavioral goal monitoring for the American Diabetes Association (ADA)–recognized DSMES Programs [7,8]. Chronicle Diabetes enables educators and their patients to set collaborative goals for their diet and physical activity; however, the lack of an interface to attach a diary was perceived to be one of the barriers by educators [9]. Directly connecting patient’s goal-setting and self-monitoring information collected through smartphones to Chronicle Diabetes can facilitate education processes so that educators can better coordinate with care plans and efficiently deliver a potentially more effective and tailored intervention.

We used the Chronicle Diabetes system currently freely available to diabetes educators through ADA–recognized DSMES programs to set patients’ diet and physical activity goals and to integrate patients’ self-monitoring information collected from smartphones and wearable fitness trackers to improve educators’ ability to monitor patients' adherence to goals. Moreover, using a central location for tracking patients’ behaviors and progress enables long-term self-management support for sustained behavior change. A system with good usability should foremost have a functionality design that matches the work domain and a user interface that supports efficient task performance by the users. We used a usability framework developed to ensure high usability of connected health systems to guide the development of our proposed connected system [10].

Our study developed such an interface and tested its usability, acceptability, and feasibility in a multi-site randomized clinical trial. The objective of this paper is to report on a study protocol for a pragmatic multi-site trial and the intervention components, including the detailed connected health interfaces.

## Methods

### Sample

Eligibility was assessed prior to participant enrollment. To participate in the study, individuals had to have been diagnosed with T2DM, be 18 years or older, own a smartphone compatible with the Jawbone UP24 fitness tracker, and be overweight or obese as classified by their body mass index >25kg/m^2^. A list available on the Jawbone website was used to determine whether participants’ smartphones were compatible with the Jawbone fitness tracker. Research assistants also searched the smartphones’ respective app stores to determine whether the companion app to the Jawbone UP24, *UP,* could be downloaded and whether the fitness band could be synced to the phone. Individuals whose smartphones were not compatible with the *UP* app or Jawbone fitness tracker were ineligible for participation in the study. Overweight or obesity status was assessed using self-reported height and weight. In addition, individuals undergoing treatment for severe psychiatric illness were not eligible to participate in the study; however, no potential participants were deemed ineligible based on this criterion.

### Sample Size Justification

We enrolled 60 patients for the study: 30 in Houston and 30 in Pittsburgh. In a national study testing the behavioral lifestyle intervention in a nontranslational setting [2], hemoglobin A_1c_ (HbA_1c_; %) levels dropped from 7.29 to 7.15 after one year in the standard intervention group, whereas the levels dropped more sharply, from 7.25 to 6.61, in the intensive intervention group. The standard deviations were 1 in each group and each repeated measurement. Assuming a correlation of 0.8 between repeated measurements and alpha=.05, we estimated that enrollment of at least 27 patients per group would result in power > 80% to detect the interaction between group and time (pre- and postintervention). The power was estimated by simulating 1000 normal samples based on observed means and variances of the trial. Allowing for 10% attrition at the end of the 3-month follow-up, we sought to enroll 30 patients in each group.

### Recruitment, Screening and Enrollment

Individuals were recruited from ADA–recognized DSMES programs in Houston, Texas and Pittsburgh, Pennsylvania. Patients were asked if they had a smartphone and whether they would be interested in participating in a research study in which they would monitor their diet and physical activity using a fitness tracker and smartphone app. Those who expressed interest were provided with more detailed information about the study and screened for eligibility. Informed consent was provided by all eligible patients. This study was approved by the Institutional Review Boards of The University of Texas Health Science Center at Houston and the University of Pittsburgh.

### Randomization

Eligible patients were randomly assigned to the intervention program or a standard diabetes education program in a 1:1 allocation ratio. The study statistician created a randomization sheet to randomly assign the patients at the time of enrollment after written informed consent was obtained.

### Treatment Procedures

#### Standard Diabetes Education Group

The recruiting sites all offer ADA–recognized diabetes education programs. During the study, patients in the standard diabetes education group saw their diabetes educators at baseline and for the follow-up data collection visit at 3 months. The patients’ interaction with their diabetes educators included setting and modifying patients’ goals related to nutrition, physical activity, risk prevention, self-monitoring of blood glucose, and medication based on their self-report of their progress. Additional visits could be scheduled as usual care based on patients’ conditions. These visits were recorded as confounding factors that would indicate any treatment or patient condition changes during the study period.

#### Connected Group

Participants in the connected group received standard diabetes education as described above. In addition, participants randomly assigned to this group were exposed to the following intervention components and procedures:

- *Goal Setting:* Diabetes educators and patients mutually set nutrition and physical activity goals and established plans for achieving the goals they documented in the Chronicle Diabetes system, in addition to the DSMES content that diabetes educators typically deliver, depending on participants’ conditions.
- *Self-Monitoring:* During the baseline visit, each study participant received a Jawbone UP24 wristband with a companion smartphone app for monitoring their diet and physical activity behaviors according to the goals set during the education visit. The study team assisted patients with creating user accounts for device use. In addition, patients received printed instruction manuals and access to YouTube tutorial videos to orient them to the Jawbone device and the companion app. Participants received hands-on training on how to self-monitor their diet and physical activity habits using the Jawbone UP24 at the beginning of the study. Specifically, patients were instructed to record their physical activity and foods eaten using the Jawbone UP24 app and asked to wear the Jawbone wristband for step tracking on a daily basis for 3 months. Participants practiced entering a meal and a workout into the smartphone app, as well as editing each of these entries. Food intake was to include all items and portion sizes consumed in a given meal, including any condiments used. The smartphone app automatically calculated calories, grams of fat, and carbohydrates for each meal, given the portions sizes entered were correct. Any exercise, including the type, duration, and level of intensity, was to be recorded daily as well. Using participants’ height and weight data, calories burned were automatically calculated, given the type, duration, and intensity of exercise were entered correctly. Participants were encouraged to log their dietary behaviors and physical activity in real-time whenever possible so that calorie totals would be accurate and so that they could make adjustments to their food choices and level of physical activity throughout the day.

### Jawbone UP24 fitness tracker and its Companion Smartphone App

The Jawbone UP24 smartphone app, *UP*, offers several ways to enter dietary behaviors and physical activity. For example, food items can be logged by searching for popular foods in the food database, scanning the barcodes on packaged items, and selecting from a restaurants’ menu. *UP* stores nutritional information for thousands of foods and gives each food item a score (1-10) to help users know which foods are most and least healthy. In addition, the fitness tracker has a recognition feature to automatically detect whether an individual is doing some type of exercise. When exercise is detected, the smartphone app asks whether a workout was completed. However, if a workout is not detected, the user can still log an exercise session. Regardless of if a workout is detected or entered into the smartphone app, the fitness tracker wristband that the user is wearing is continually tracking the number of steps that the user is taking. All data from the fitness tracker wristband are wirelessly synced to the smartphone app using Bluetooth technology.

### Chronicle Diabetes System

**Figure 1:**
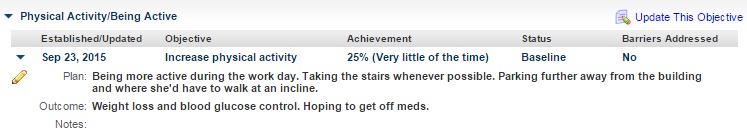
A partial snapshot of the Chronicle Diabetes system with goal setting function.

According to the national standards for DSMES, diabetes educators are expected to establish and track patients’ behavioral goals [11]. Chronicle Diabetes (http://www.chronicle diabetes.com) is a Health Insurance Portability and Accountability Act (HIPAA)–compliant Web-based electronic diabetes education system developed by the University of Pittsburgh and adopted for reporting outcomes for the ADA–recognized diabetes education programs. All ADA–recognized diabetes education programs have free access to Chronicle Diabetes. During an education session, the patients and diabetes educators can mutually initiate behavioral goals and diabetes educators can use the Chronicle Diabetes system to document the goals by selecting from one of the seven self-care goal categories: healthy eating, being active, monitoring, taking medication, problem-solving, reducing risks, and healthy coping. Patients’ goal achievement can be scored at 0%, 25%, 50%, 75%, and 100% at baseline. In addition, diabetes educators and their patients can choose to continue, modify, or discontinue the goals at follow-up visits. This allows for a patient’s progress towards meeting a goal to be tracked over time (Figure 1). In our previous evaluation of the Chronicle Diabetes system, the preliminary analysis showed that the diabetes educators favored the feature of setting behavioral goals, the majority of which were focused on nutrition and physical activity [7,8].

**Figure 2:**
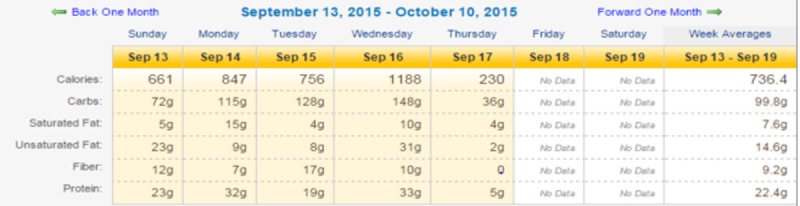
Partial Screenshot of Monthly Calendar View of Diet Monitoring in Chronicle Diabetes.

While educators found Chronicle Diabetes useful for setting and tracking behavioral goals, one of the major barriers they identified was the lack of a feature to attach a food diary for educators to evaluate patients’ adherence to the prescribed goals. Thus, in this study, we developed a connected interface in the Chronicle Diabetes System to connect data from the Jawbone UP24 fitness tracker and its companion smartphone app for patient diet and physical activity data. Within each patient’s documentation record in Chronicle Diabetes, two new sections (tabs) of the record were added: “Self Monitoring–Nutrition” and “Self Monitoring–Activity.” These two tabs were used by educators to navigate to two pages showing self-monitored diet and activity information. When educators click the “Self Monitoring–Nutrition” tab in the navigation panel for a patient for the first time, a link to connect to the patient’s account will appear. In the beginning of the study, educators needed to click a link in this page to connect the patient’s Jawbone account to their account in the Chronicle Diabetes system. The link took the educator into a page to select what they wanted to connect to from a list of the devices or apps that were supported by Validic, an intermediary platform that provides connection to data from a wide variety of devices and apps. In our study, educators were instructed to select Jawbone and enter their account information to complete the connection process. The link can also be sent to participants via a previously generated email template. The educator can encourage the patient to complete the connection process during the first visit after enrolling in the study. Once the connection process is complete, a monthly calendar view will appear in the “Self Monitoring–Nutrition” page with cells for calories, carbs, saturated and unsaturated fat, fiber, and protein for each day (See Figure 2). The weekly average of these macronutrients is also available in the far-right column of this calendar. Educators can click on any single day to view the food types, macronutrient information broken down by meal, and time of the meal.

**Figure 3:**
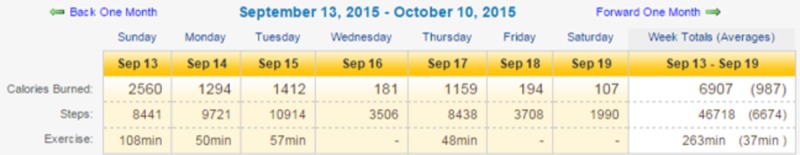
Partial Screenshot of Monthly Calendar View of Activity Monitoring in Chronicle Diabetes.

The “Self Monitoring–Activity” page has the same monthly calendar view as the “Self Monitoring–Nutrition” page once connected: calories burned, steps, and planned exercise duration for each day (Figure 3), along with the weekly total and average for these 3 activity parameters. Educators can click on each day for more details about patients’ activity, including the time, type, and intensity levels of the activities (Figure 3). In this connected interface, educators can switch between the nutrition and physical activity pages easily by clicking on the two tabs on the left (the month being viewed on one tab is automatically displayed on the other tab, allowing for easy correlation between the two months). Also, they can use the “Jump to date” function to select a particular date of interest and can go forward and back one month in the calendar view to quickly review data over a few months and see trends.

### Validic as an Intermediary Platform Between Mobile Apps and Devices and the Chronicle Diabetes System

We used Validic as an intermediary platform to connect the Jawbone UP24 diet and physical activity data to the Chronicle Diabetes system. We adopted this approach rather than directly connecting mobile data and Chronicle Diabetes owing to 1) the flexibility of connecting to additional fitness tracking systems in the future, 2) robust screening of data and high-level security through Validic, and 3) Validic’s experience working with large EHR systems. We believe the incorporation of Validic not only enables a seamless transition for national dissemination of our developed connected interface but also enables its future integration with EHRs seamlessly to make connected technology available to any health care system using an EHR system.

### Data Security in Smartphone Use, Transit, and the Chronicle Diabetes System

The security features are based on the premise that any data at rest or in motion must be encrypted and unreadable to outsiders. User profile, clinical data, and progress stored in the local SQL lite database on the smartphones are encrypted using Advanced Encryption Standard/Rivest-Shamir-Aldeman algorithms. When the data are sent to the server, the data are encrypted using the session ID received from the server in its initial authorization token. This encrypted data is sent over HTTPS, thus allowing 2 levels of security. When the data reach the server, they are decrypted using the session identification and stored in the database by encryption again using a server-specific encryption algorithm. Thus, the basic premise is satisfied and the data are completely secure when collected through smartphones, in transit, and when accessed from the Chronicle Diabetes system. All data from the Chronicle Diabetes system are stored securely in an HIPAA–compliant manner and restricted based on access privileges. An HIPAA Business Associates Agreement is signed with each facility using this system. All data access is recorded and a full audit trail can be produced.

### Outcome Measures

#### Feasibility

Feasibility of the study was evaluated through participant attrition rates and qualitative and quantitative assessments of the usability of the connected system. Usability of the connected interface technology was measured using the System Usability Scale [12]. The System Usability Scale measures patients’ and educators’ acceptability, satisfaction, and perceived usefulness of the intervention that is the focus of the study (that is, the connected interface technology within Chronicle Diabetes). Patients and educators were asked to rate each usability item in the scale from 0 to 100.

#### Preliminary Efficacy

Preliminary efficacy was measured by changes in patients’ HbA_1c_ levels and weight from baseline to 3 months. HbA_1c_ levels were measured using the *A1cNOW* self-check system via a finger stick or extracted from clinical visit data. Participants’ weight was measured via a weight scale in the clinic or self-report.

#### Additional Baseline and Follow-Up Measures

At baseline, a sociodemographic and medical history questionnaire is used to collect study participants’ socio-demographic information, diabetes treatment plan, and other medical history. At 3 months, we asked patients how many medical, emergency, and diabetes education visits were made in between the 2 study visits and recorded this information as potential confounding factors of the study outcome.

### Data Analysis Plan

SAS (version 9.2, SAS Institute, Inc, Cary, NC) was used for data screening and analyses. All the statistical tests were performed at 5% level-of-significance. The outcome variables HbA_1c_ (%) level and weight change were used to measure the preliminary efficacy of this study. Qualitative thematic analysis was used to analyze interview data.

## Results

We recruited 30 patients from Houston, Texas and 30 patients from Pittsburgh, Pennsylvania, through various diabetes education programs recognized by the ADA. At each site, 30 enrolled patients were randomly assigned to the intervention group or standard diabetes education group. Data collection is completed. Data analysis is in progress. The study results will be reported in mid-2018.

## Discussion

This study leveraged existing DSMES programs, resources, and diabetes educators to deliver the technology-based program. Although mobile health interventions were developed to improve patient self-management in various research efforts [13-15], none of these efforts focused on using data from mobile devices for clinicians to use in clinical practice. The implementation of the evidence-based behavioral goal setting and monitoring program in the diabetes education setting using technology provides an opportunity to secure reimbursement for delivering the program in a practice setting. This study helped us test the implementation of the evidence-based behavior intervention using a newly developed interface-connected technological assistance built on an existing EHR system used by diabetes educators to facilitate the long-term implementation in a diabetes education setting in a 3-month randomized controlled trial with ADA–recognized diabetes education programs.

Using smartphones and connected wearable fitness trackers not only reduces the burden of patient self-monitoring but also enables the connection of daily patient information to the Chronicle Diabetes clinical information system, where educators can track patients’ behaviors between visits in a graphical format and prepare for the next intervention session. This connection could also serve as an interactive platform to deliver intervention and provide feedback from diabetes educators in real time in the future. Connection to a national recognition data base through Chronicle Diabetes also offers the potential to collect behavioral and clinical information on unique populations and practices nationwide. Connecting patients’ self-monitoring information collected through smartphones to Chronicle Diabetes can facilitate education processes by allowing educators to more efficiently coordinate care plans with patients and ensure delivery of effective and tailored interventions. Moreover, using a central location for tracking patients’ behaviors and progress enables long-term self-management support for sustained behavior change. A system with good usability should foremost have good design of functionality that matches the work domain and a user interface that supports efficient task performance by the users [10]. The usability evaluation conducted in this study could also provide scientific evidence and support for other noncommunicable diseases that may benefit from continuous behavior monitoring. The interface developed in this study could also be used for more interactive designs in future studies, such as enabling educators to send tailored feedback to patients’ smartphones.

This study could be easily and widely disseminated in future studies and practice. There are increasing numbers of smartphone users in the United States, including minority populations. As ADA–recognized education programs with access to the Chronicle Diabetes system are located throughout the US, this study could be widely disseminated. We anticipated that diabetes educators would use this connected tool to engage diabetes patients in lifestyle changes in between diabetes education visits and facilitate conversation on meeting or changing behavioral goals at follow-up diabetes education visits, rather than use data from the connected tool as a stand-alone piece of information to make treatment changes. The smartphone and wearable tracker usage in this study is only a tool to assist with diabetes educators, not to replace the role of a diabetes educator. The wearable fitness tracker and its companion smartphone app are not approved by the US Food and Drug Administration and their accuracy on measuring physical activity levels and dietary information is not guaranteed. Thus, the data connected from these devices and smartphone apps to Chronicle Diabetes system should be used with caution.

In summary, if proven effective, the study will not only advance nursing and behavioral science by leveraging existing resources to disseminate an evidence-based behavior intervention via emerging technology, but it will also provide theoretical and methodological guidance for other researchers conducting usability evaluations connecting mobile device information collection with EHR systems for managing diabetes and other chronic conditions.

## Abbreviations

ADA: American Diabetes Association
DSMES: diabetes self-management eucation and support
EHR: electronic health record
HIPPA: Health Insurance Portability and Accountability Act
T2DM: type 2 diabetes mellitus
